# The Adipose Microenvironment Dysregulates the Mammary Myoepithelial Cells and Could Participate to the Progression of Breast Cancer

**DOI:** 10.3389/fcell.2020.571948

**Published:** 2021-01-11

**Authors:** Laetitia Delort, Juliette Cholet, Caroline Decombat, Marion Vermerie, Charles Dumontet, Florence A. Castelli, François Fenaille, Céline Auxenfans, Adrien Rossary, Florence Caldefie-Chezet

**Affiliations:** ^1^Université Clermont Auvergne, INRAE, UNH, ECREIN, Clermont–Ferrand, France; ^2^Université Lyon 1, INSERM U1052, CNRS 5286, Cancer Research Center of Lyon, Lyon, France; ^3^Université Paris-Saclay, CEA, INRAE, Département Médicaments et Technologies pour la Santé (DMTS), MetaboHUB, Gif-sur-Yvette, France; ^4^Banque de Tissus et de Cellules, Hôpital Edouard-Herriot, Lyon, France

**Keywords:** myoepithelial cells, breast cancer progression, obesity, human adipose stem cells, mature adipocytes, miRNA, metabolomics

## Abstract

Breast cancer is the most common cancer among women worldwide. Overweight and obesity are now recognized as established risk factors for this pathology in postmenopausal women. These conditions are also believed to be responsible for higher recurrence and mortality rates. Reciprocal interactions have been described between adipose and cancer cells. An adipose microenvironment favors a greater proliferation of cancer cells, their invasion and even resistance to anti-cancer treatments. In addition, the chronic low-grade inflammation observed in obese individuals is believed to amplify these processes. Among the cell types present in the breast, myoepithelial cells (MECs), located at the interface of the epithelial cells and the stroma, are considered “tumor suppressor” cells. During the transition from ductal carcinoma *in situ* to invasive cancer, disorganization or even the disappearance of MECs is observed, thereby enhancing the ability of the cancer cells to migrate. As the adipose microenvironment is now considered as a central actor in the progression of breast cancer, our objective was to evaluate if it could be involved in MEC functional modifications, leading to the transition of *in situ* to invasive carcinoma, particularly in obese patients. Through a co-culture model, we investigated the impact of human adipose stem cells from women of normal weight and obese women, differentiated or not into mature adipocytes, on the functionality of the MECs by measuring changes in viability, apoptosis, gene, and miRNA expressions. We found that adipose cells (precursors and differentiated adipocytes) could decrease the viability of the MECs, regardless of the original BMI. The adipose cells could also disrupt the expression of the genes involved in the maintenance of the extracellular matrix and to amplify the expression of leptin and inflammatory markers. miR-122-5p and miR-132-3p could also be considered as targets for adipose cells. The metabolite analyses revealed specific profiles that may be involved in the growth of neoplastic cells. All of these perturbations could thus be responsible for the loss of tumor suppressor status of MECs and promote the transition from *in situ* to invasive carcinoma.

## Introduction

Breast cancer is the most common cancer among women, with more than 2.1 million new cases around the world and more than 626,000 deaths (Ferlay et al., 2019). Epidemiological studies have confirmed the association between overweight and obesity with a higher risk of postmenopausal breast cancer (*RR* = 1.12, 95% confidence interval [95% CI] = 1.09–1.15) (World Cancer Research Fund [WCRF], 2017), larger tumors, positive lymph-node status and reduced outcomes, regardless of menopausal status (Dignam et al., 2003; Picon-Ruiz et al., 2017). To understand the link between obesity and breast cancer, a large number of studies have been focused on the adipose tumor microenvironment and showed its importance in the development, growth and progression of cancer. Reciprocal interactions have been revealed between breast cancer cells and their adipose microenvironment, particularly in obese situations. The adipose microenvironment, mainly consisting of adipocytes as well as adipose stem cells, fibroblasts, endothelial and immune cells, is able to promote angiogenesis (Bougaret et al., 2017; Quail and Dannenberg, 2019; Yadav et al., 2020) and to reduce the efficiency of anticancer treatments, such as tamoxifen (Bougaret et al., 2018; Delort et al., 2019). Adipose tissue is considered to be an endocrine organ capable of secreting soluble factors (growth factors, cytokines, adipokines, proteases and vascular stimulation factors) that can act on surrounding cells and on the composition of the extracellular matrix (Walter et al., 2009). The mammary stroma can undergo phenotypic and functional changes in order to be active and provide a favorable environment for the development of the mammary tumor (Place et al., 2011). Knowledge of the underlying pathophysiological mechanisms could be beneficial in the management of overweight patients.

Breast tissue is composed of myoepithelial cells (MECs). These cells are little studied mammary cell types but which play a major role in the normal structure of the breast. MECs are hybrid of both smooth muscle and epithelial cells that are present around the acini of certain exocrine glands, such as the mammary glands and whose contraction results in the expulsion of the secretion product from the glandular acini (Adriance et al., 2005). These cells play a role in basement membrane formation and lactation through the expression of type IV collagen, laminin, actin and oxytocin receptors. MECs constitute an almost continuous layer of cells that surround luminal epithelial cells, separating them from the basement membrane and the stroma and limiting tumor invasion. MECs can be detected by specific markers (p63, CD10, α-smooth muscle actin) and constitute a major criterion for pathologists to distinguish between ductal carcinoma *in situ* (DCIS), for which epithelial cells are separated from the stroma by this continuous layer of MECs, and invasive ductal cancer (IDC), for which cancer cells are in direct contact with stroma cells (Allinen et al., 2004; Polyak and Hu, 2005). Data suggest that MECs are critical for the maintenance of DCIS and the invasion process (Sirka et al., 2018).

MECs are referred to as “tumor suppressors.” In addition to forming a physical barrier against the invasion of tumor cells, studies have shown that they strongly express extracellular matrix proteins (collagen, laminin A, fibronectin, osteonectin, etc.), proteins involved in angiogenesis (thrombospondin-1, plasminogen) and protease inhibitors (Polyak and Hu, 2005). They have the ability to inhibit the growth, invasion and angiogenesis of breast cancer cells, this means the loss of CD44 and the expression of protease inhibitors. These cells also have anti-angiogenic, anti-proliferative and anti-invasive properties through the expression of proteins known for their tumor suppressing effect (p63, p73, maspine). They are also involved in the polarity of the luminal epithelial cells *via* specific desmosomal proteins and laminin-1 release (Allen et al., 2014).

Modifications of gene expression have been observed in the case of a DCIS compared to a normal myoepithelium. Proteases (cathepsin F, K, L; MMP2, PRSSAA), protease inhibitors (thrombospondin-2, SERPIN-1, cystatin C, TIMP3) and collagen are strongly overexpressed at the MEC level in the case of DCIS, suggesting a major role for these cells in extracellular matrix remodeling (Allinen et al., 2004). Our team has already demonstrated that MECs express high levels of adiponectin in breast tissue adjacent to tumor samples, an adipokine known to have antiproliferative activities, and to not express leptin, an adipokine with procarcinogenic properties. Thus, these cells, through adiponectin secretion, would have the ability to protect normal epithelial cells and inhibit the proliferation of breast cancer cells through a paracrine effect (Jarde et al., 2008). Co-culture experiments have shown that MECs are able to decrease the expression of metalloproteinases by mammary cancer cells, even in the presence of fibroblasts, thus confirming the dominance and the importance of these MECs in invasion. In addition, these cells have increased anti-proliferative and anti-invasive capacities in the presence of tamoxifen, following an increase in maspine secretion, and in the production of inducible nitric oxide synthase (iNOS). These effects are reportedly mediated by the estrogen receptor (ER), as MECs express ERα and not ERβ (Jones et al., 2003). Few studies have focused on the impact of obesity on MECs. Chamberlin et al. (2017) have shown that obesity was able to increase the ratio of luminal to basal/myoepithelial cells in both human samples and a model of obese mice, confirming the positive correlation between increased BMI and the reduced number of MECs. Their model of obese mice revealed that the continuous layer of MECs in normal mammary gland was discontinued in obese mice and similar observations were made in human samples (Chamberlin et al., 2017).

For all these reasons, we hypothesized that the mammary adipose tumor microenvironment could be involved in the functional modifications of MECs, reducing/inhibiting their tumor-suppressive capacities and disrupting the spatial organization of MECs. This would lead to favor the invasion of cancer cells and the transition from DCIS to IDC, particularly in an obese situation. Using a co-culture model, we studied the impact of human adipose cells from women of normal weight and obese women on the functionality of the MECs by measuring their impact on viability, apoptosis and gene expression. The role of miRNAs was also evaluated in our model. Our study revealed that adipose cells (adipose precursors and mature adipocytes) could be able of modifying the functional characteristics of the MECs and these effects could be mediated by leptin and inflammatory markers.

## Materials and Methods

### Co-culture Experiments

#### Cell Culture

##### Myoepithelial Cells (MECs)

Hs578Bst normal myoepithelial breast cell line was obtained from ATCC and cultured in DMEM/F12 (Gibco, Thermo Fisher Scientific, Waltham, United States) supplemented with EGF (30 ng/mL, Sigma-Aldrich, Saint Quentin Fallavier, France), fetal bovine serum (10%), L-glutamine (1%) and gentamycin (50 μg/mL) (Thermo Fisher Scientific).

##### Adipose Cells

*The human multipotent adipose cell line (hMAD)* is a kind gift from Charles Dumontet (University Lyon 1, Lyon, France; INSERM U1052, CNRS 5286, Cancer Research Center of Lyon, Lyon, France). *Human adipose stem cells (hASCs)* were kindly provided by the Cell and Tissue Bank (Hôpital Edouard-Herriot, Lyon, France). hASCs were obtained from patients undergoing surgery for cosmetic purposes without associated pathology according to Helsinki declaration from anonymous healthy donors. Surgical residue was harvested according to French regulation including declaration to research ministry (DC n°2008162) and procurement of written informed consent from the patient. hASCs were extracted from subcutaneous adipose tissue from women undergoing optimized liposuction and who were of normal weight (hASC20: human adipose stem cells from women of normal weight) or obese (hASC30: human adipose stem cells from obese women). hASCs were extracted (Mojallal et al., 2008) using a 3 mm cannula according to ethical and safety guidelines as approved by the local IRB and as described by Björntorp (Lequeux et al., 2009). Four different strains were obtained from women of normal weight (BMI comprised between 18 and 22) and 6 from obese women (BMI > to 30).

##### Differentiation of hASCs

hMAD cells and hASCs were seeded at confluence (33,500 cells/cm^2^) in a differentiation medium consisted of DMEM/F12 (1:1) supplemented with 10% FBS, hydrocortisone (25 μg/mL), insulin (3.5 mg/mL), T3 (6.5 μg/mL), dexamethasone (980 μg/mL), rosiglitazone (1.78 mg/mL), IBMX (100 mg/mL, only the first 3 days), gentamycin (50 μg/mL). The medium was replaced every 2 days. Mature adipocytes (MAs) were obtained after 12 days of differentiation (hMAD#, MA20, MA30) (Bougaret et al., 2017). The effectiveness of differentiation as well as the ability of hASCs to maintain a metabolism representative of their tissue of origin (collected from women of normal weight or obese women) has been previously validated (Bougaret et al., 2017).

All the cells used were under mycoplasma-free conditions and cultured in a 5% CO_2_-humidified incubator at 37°C.

#### Co-culture Between MECs and Adipose Cells

Interactions between MECs and adipose cells were evaluated using a co-culture system (Transwell culture system, porosity 0.4 μm). MECs were seeded at the bottom of wells (5,000 cells/well) and co-cultured with either hASCs (hMAD cells or hASCs) seeded in inserts (5,000 cells/inserts) or hASCs previously differentiated into MAs (hMAD#, MA) in DMEM/F12 medium supplemented with FBS (10%) and glutamine (1%). After 72 h of incubation, the culture medium was substituted with a resazurin solution (25 μg/mL), which in the presence of metabolically active cells is oxidized to a pink fluorescent resorufin product and whose fluorescence intensity is proportional to the number of viable cells (Exw = 530 nm and Emw = 590 nm, Fluoroskan Ascent FL^®^, Thermo Fisher Scientific). Experiments were realized at least three times for the study of hMAD cell line and at least eight times for the hASCs and MAs. Results were expressed as percentage of growth ± SEM.

#### Culture With Conditioned Media (CM)

Conditioned media (CM) were collected after 48 h of culture of hASCs or MAs in DMEM/F12 supplemented with FBS (10%) and glutamine (1%) and kept under nitrogen atmosphere at −80°C. To assess the specific role of adipose secretome, MECs were exposed to CM (dilution 1:1 in fresh complete adipose cell media) and the viability was measured after 72 h with the resazurine test (3 independent experiments) (Bougaret et al., 2018).

#### Apoptosis Assay

Following viability assays, MECs were washed with phosphate buffered saline (PBS), recovered by centrifugation at 1,000 g for 5 min at room temperature (RT), and resuspended in 40μL of annexin V binding buffer (140 mm NaCl, 10 mm HEPES/NaOH, 2.5 mm CaCl_2_). The cell suspension was stained with 5 μL of annexin V-FITC and 5 μL of propidium iodide (PI), and incubated for 15 min at RT in the dark. After the addition of PBS and a centrifugation at 1,000 g for 5 min at RT, cells were resuspended in 50 μL of annexin V binding buffer. Stained cells were then analyzed on a Cellometer K2 image cytometer (Nexcelom Bioscience, Lawrence, MA, United States). Data were expressed as percentage of live cells, % cells in early or late apoptosis and % of necrotic cells. Experiments were realized three times.

#### Quantitative Real-Time PCR (qPCR) Assays

Total RNA was extracted from co-cultured or not MECs with TRIZOL reagent (Invitrogen, Thermo Fisher Scientific). After the evaluation of the quantity and purity (NanoDrop 2000, Thermo Fisher Scientific), DNase treatment (DNase I Amplification grade, Invitrogen) and cDNAs retrotranscription (HighCap cDNA RT Kit RNAse inhib, Invitrogen) were made according to the manufacturer’s recommendations. qPCR were performed on plates designed by Applied Biosystems (TaqMan^®^ Array 96 well Fast Plate, Customformat 48) using SDS7900HT automaton (Applied Biosystems, Thermo Fisher Scientific) with TaqMAN^®^ (Applied Biosystems). The analysis was conducted on 44 genes and 4 references genes with TaqMAN^®^ Array Fast Plates (18S, GAPDH, HPRT1, GUSB, LEP, LEPR, ADIPOQ, ADIPOR1, ADIPOR2, ESR1, ESR2, CDH1, MMP2, MMP3, MMP9, MMP14, IL6, TNF, VEGFA, MYC, AKT1, BAX, CCND1, TP53, CYP19A1, CTNNB1, PTEN, TP63, SERPINB5, TIMP1, SERPINE1, THBS1, FGFR1, MMP10, TGFB1, HSPG2, LAMA1, LAMB1, KRT5, VIM, FN1, PTGS2, CXCL12, MYBL2, CASP9, CCNB1, WT1, MKI67). Genes were considered significantly expressed and their transcript measurable if their corresponding Ct value was less than 35. Each sample was normalized to endogenous reference genes. The relative quantification method (*RQ* = 2^–ΔΔCT^) was used to calculate the relative gene expression of given samples with ΔΔCT = [ΔCT (sample1) −ΔCT (sample2)] and ΔCT = [CT(target gene) – geometric mean CT(reference genes)]. Paired *t*-tests were used for comparisons of gene expression levels with at least two valid pairs of values. Control of the false discovery rate due to multiple testing was done according to Benjamini-Hochberg method for each comparison separately. Using ΔCT values, gene expression was plotted as a heatmap, paired with a two-way hierarchical cluster analysis (package “gplots”) in R version 3.5.0. Three independent experiments were performed.

#### MiRNAs

miR expression was determined using TaqMan^®^ Advanced miRNA Assays (SDS7900HT Fast Real-Time PCR Systems, Applied Biosystems, Thermo Fisher Scientific) according to the manufacturer’s recommendations. miRNAs were first extracted with NucleoSpin miRNA and RNA purification (Macherey−Nagel, Dürer, Germany). All other steps were performed using the TaqMan^®^ Advanced miRNA Assays. Briefly, miRNAs were modified by extending the 3’ end of the transcript through a poly(A) addition, then lengthening the 5’ end by adaptor ligation. Then modified miRNAs were reverse transcribed (TaqMan^®^ Advanced miRNA cDNA Synthesis Kit, Life Technologies) followed by an amplification to increase uniformly the amount of cDNA for all miRNAs (5 min at 95°C, followed by 14 cycles with 3 s at 95°C and 30 s at 60°C and finally 10 min at 99°C). The PCR was then performed (10 min at 92°C, followed by 40 cycles with 1 s at 95°C and 20 s at 60°C). miRNAs were considered significantly detected if their corresponding Ct value was less than 32. Two exogenous controls (ath-miR159a and cel-miR-39-3p) non expressed in human permitted to validate experiments. A global mean normalization was used to normalize data. Gene expression was measured using the comparative Ct (ΔΔCt) method of relative quantitation. The analysis workflow was made by DataAssist software (Applied Biosystems). Four independent experiments were performed. Using DCT values, gene expression was plotted as a heatmap, paired with a two-way hierarchical cluster analysis (package “gplots”) in R version 3.5.0. The predicted miRNA target were determined using mirPath v.3 from DIANA TOOLS bioinformatics resources through gene ontology biological process (GO) terms and The Kyoto Encyclopedia of Genes and Genomes (KEGG) pathway terms (Vlachos et al., 2015).

## Untargeted Metabolomics by Liquid Chromatography Coupled to High-Resolution Mass Spectrometry

In order to identify the molecules potentially responsible for the effect on the MECs, the analysis of culture media of hASCs and MAs differentiated from hASCs from women of normal weight and obese women was carried out by mass spectrometry-based untargeted metabolomics.

### Chemicals and Reagents

All analytical grade reference compounds were from Sigma-Aldrich (Saint Quentin Fallavier, France). The standard mixtures used for the external calibration of the spectrometer (Calmix-positive, for the positive ion mode, consisting of caffeine, L-methionyl-arginyl-phenylalanyl-alanine acetate, and Ultramark 1621, and Calmix-negative, for the negative ion mode, consisting of the same mixture plus sodium dodecyl sulfate and sodium taurocholate) were from Thermo Fisher Scientific (Courtaboeuf, France). Acetonitrile (ACN) was from SDS (Peypin, France), formic acid from Merck (Briare-le-Canal, France), methanol from VWR Chemicals (Fontenay-sous-Bois, France) and deionized water from Biosolve chemicals (Dieuse, France).

### Metabolite Extraction

After thawing at room temperature, culture cell supernatants were immediately processed. Samples (50 μL) were deproteinized in microcentrifuge tubes by adding 200 μL of methanol, thoroughly vortexed, incubated in an ultrasonic bath for 5 min and then left on ice for 90 min. After vortexing, the samples were centrifuged at 20,000 g for 15 min at 4°C. The supernatant was then evaporated to dryness under a nitrogen stream at 30°C using a Turbovap (Caliper Life Science Inc., Roissy, France). Then, 150 μL of either H_2_O:ACN (95:5, v/v), with 0.1% formic acid or 10 mM ammonium carbonate pH 10.5:ACN (40:60, v/v) were added to the residue to reconstitute the culture cell extracts for RP and HILIC analyses (see below), respectively. The tubes were vortexed again and incubated in an ultrasonic bath for 5 min and centrifuged for another 10 min. A volume of 95 μL of the supernatant was transferred to 0.2 mL vials. Internal standard solution (5 μL; mixture of 9 authentic chemical standards covering the mass range of interest: 13C-glucose, 15N-aspartate, ethylmalonic acid, amiloride, prednisone, metformin, atropine sulfate, colchicine, imipramine) was added to all samples in order to check for consistency of analytical results in terms of signal and retention time stability throughout the experimental batch. In addition, a quality control (QC) sample was obtained by pooling 20 μL of each sample and was injected every five samples in order to evaluate the signal variations of each metabolite.

### Liquid Chromatography Coupled to High-Resolution Mass Spectrometry (LC-HRMS)

Metabolite profiling was performed using a combination of two complementary chromatographic columns, a Hypersil GOLD C8 (RP, 1.9 μm, 2.1 mm × 150 mm column, Thermo Fisher Scientific), and a Sequant ZICpHILIC 5 μm, 2.1 × 150 mm (HILIC) (Merck, Darmstadt, Germany) that were maintained at 30°C and 15°C, respectively. Both columns were equipped with an on-line prefilter (Thermo Fisher Scientific). Experimental settings for each LC-HRMS condition are described below.

Mobile phases for the RP column were 100% water in A and 100% ACN in B, both containing 0.1% formic acid. Regarding HILIC, phase A consisted of an aqueous buffer of 10 mM ammonium carbonate in water adjusted to pH 10.5 with ammonium hydroxide, whereas pure ACN was used as solvent B. Chromatographic elutions were achieved under gradient conditions as follows:

(i) RP-based system: the flow rate was set at 500 μL/min. The elution consisted of an isocratic step of 2 min at 5% phase B, followed by a linear gradient from 5 to 100% of phase B for the next 11 min. These proportions were kept constant for 12.5 min before returning to 5% B for 4.5 min.
(ii) HILIC-based system: the flow rate was 200 μL/min. Elution started with an isocratic step of 2 min at 80% B, followed by a linear gradient from 80 to 40% of phase B from 2 to 12 min. The chromatographic system was then rinsed for 5 min at 0% B, and the run ended with an equilibration step of 15 min (80% B).

LC-HRMS analyses were performed using an Ultimate U3000 liquid chromatography system coupled to an Exactive mass spectrometer from Thermo Fisher Scientific fitted with an electrospray (ESI) source and operated in the positive and negative ion modes for metabolite separations on C18 and ZIC-pHILIC columns, respectively. The software interface was Xcalibur (version 2.1, Thermo Fisher Scientific). The mass spectrometer was externally calibrated before each analysis in both ESI polarities using the manufacturer’s predefined methods and recommended calibration mixtures provided by the manufacturer.

The Exactive mass spectrometer was operated with a capillary voltage set at −3 kV in the negative ionization mode and at 5 kV in the positive ionization mode, and with a capillary temperature set at 280°C. The sheath gas and the auxiliary gas pressures were set at 60 and 10 arbitrary units with nitrogen gas, respectively. The mass resolution power of the analyzer was 50,000 at *m/z* 200 (full width at half maximum) for singly charged ions. The detection was achieved from *m/z* 85 to 1,000 for RP conditions in the positive ionization mode and from *m/z* 75 to 1,000 for HILIC conditions in the negative ionization mode.

### Data Treatment and Handling

All raw data were first manually inspected using the Qualbrowser module of Xcalibur version 2.1 (Thermo Fisher Scientific). Raw files were first of all converted to mzXML format using MSConvert software. Automatic peak detection and integration were performed using the XCMS software package (W4M platform; Giacomoni et al., 2015)^1^.

### Metabolite Annotation and Identification

Features were annotated by using our spectral database according to accurately measured masses and chromatographic retention times (Boudah et al., 2014). Confirmation of metabolite annotation was then accomplished by running additional LC-MS/MS experiments using an Ultimate chromatographic system combined with a Q-Exactive mass spectrometer (Thermo Fisher Scientific) under non-resonant collision-induced dissociation conditions using higher-energy C-trap dissociation (HCD). To be identified, ions had to match at least two orthogonal criteria (accurately measured mass, isotopic pattern, MS/MS spectrum and retention time) to those of an authentic chemical standard analyzed under the same analytical conditions, as proposed by the Metabolomics Standards Initiative (Sumner et al., 2007).

### Statistical Analyses

Chromatographic peak areas contained in the two filtered and processed XCMS data matrices (i.e., RP-UHPLC-HRMS with MS detection in positive ESI modes, and HILIC-HPLC-HRMS with MS detection in the negative ESI mode) were statistically analyzed using both multivariate (PCA, PLS-DA) and univariate statistical tests available on the W4M platform (see text footnote 1) in order to identify pertinent metabolites. The significant metabolites were imported into the freely available online MetExplore tool (Cottret et al., 2018) for metabolic pathway enrichment.

## Results

### Adipose Precursors and Differentiated Adipose Cells Are Able to Adversely Influence the Viability of Myoepithelial Cells Whatever the BMI

Our different experiments highlight the influence of adipose cells on the functionality of MECs.

**FIGURE 1:**
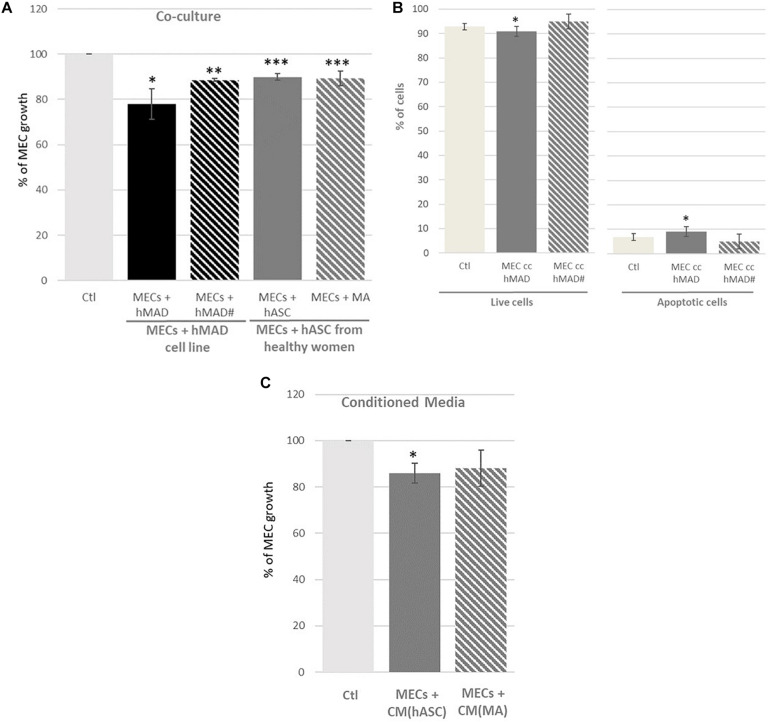
Percentage of myoepithelial cell (MEC) viability and apoptosis when they were co-cultured with adipose cells or adipose secretome. **(A)** MECs were co-cultured for 72 h with the human multipotent adipose derived cell line (hMAD) or human adipose stem cells (hASCs) extracted from healthy women and differentiated into mature adipocytes (hMAD# and MAs); **(C)** MECs were cultured for 72 h with conditioned media (CM) obtained from hASCs (CM[hASC]) or MAs [CM(MA)]. Results are expressed as Percentage of MEC growth ± SEM at 72 h of co-culture (*n* = 3 for hMAD cells, *n* = 16 for hASCs from healthy women, *n* = 3 for CM; ^∗^*p* < 0.05, ^∗∗^*p* < 0.01, ^∗∗∗^*p* < 0.001). **(B)** Evaluation of apoptosis in myoepithelial cells (MECs). MECs were co-cultured or not with non-differentiated (hMAD) or differentiated hMAD (hMAD#) for 72 h and stained with Annexin V-FITC and PI. [FITC-/PI-] reflected living cells; fluorescence [FITC+/PI-] and [FITC+/PI+] represented apoptotic cells. The percentage of cells is represented as the mean ± SEM, ^∗^*p* ≤ 0.05 compared to the control. Experiments were repeated in triplicate. Ctl, Control.

Adipose cells, whether derived from a lineage (hMAD) or from primary cells (hASCs) seemed to decrease the viability of the MECs (Figure 1A). In addition, it appears that adipocyte precursors (hMAD and hASCs) play a role as important as differentiated adipocytes (hMAD# and MAs). Indeed, when the MECs were co-cultured with hASCs (hMAD cell line or hASCs from healthy women), the viability of MECs was reduced (−22% *p* < 0.05 and −10%, *p* < 0.01 with hMAD and hASCs, respectively). Mature adipocytes (hMAD# or MAs) appeared to have a similar impact on the viability of the MECs compared to the precursors (−11%, *p* < 0.001). The evaluation of apoptosis by image cytometry (Figure 1B) showed a slight increase of the percentage of apoptotic cells when the MECs were co-cultured with hMAD. When the MECs were only exposed to the secretome (Figure 1C), the same trend was observed, with a significant decrease in the viability only in the presence of hASC secretions.

**FIGURE 2:**
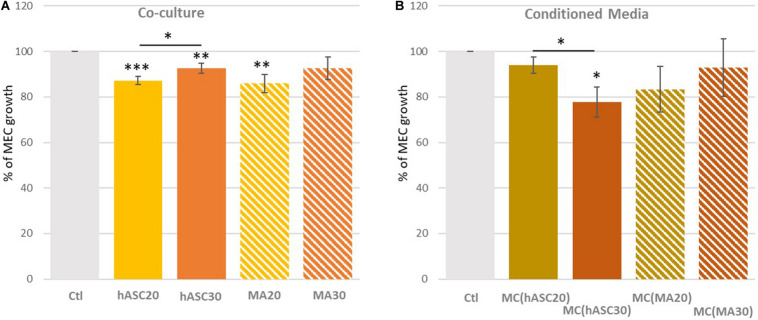
Percentage of myoepithelial cell (MEC) viability when co-cultured with adipose cells from women of different BMI **(A)** or with their adipose secretome **(B)**. **(A)** MECs were co-cultured for 72 h with human adipose stem cells (hASCs) extracted from women of normal weight (hASC20) and obese women (hASC30) and differentiated into mature adipocytes (MA20 and MA30); **(B)** MECs were cultured for 72 h with conditioned media (CM) obtained from hASCs (CM[hASC20] and CM[hASC30]) or MAs (CM[MA20] and CM[MA30). Results are expressed as Percentage of MEC growth ± SEM (*n* = 8 for hASCs and MAs, *n* = 3 for CM; ^∗^*p* < 0.05, ^∗∗^*p* < 0.01, ^∗∗∗^*p* < 0.001).

The influence of obesity was evaluated using hASCs from both women of normal weight (hASC20) and obese women (hASC30), differentiated into MAs (MA20, MA30) (Figure 2). Cells from women of normal weight and obese women, whether hASCs or MAs, appeared to decrease the viability of the MECs in the same proportions (−12% with hASC20; −8% with hASC30; −14% with MA20, *p* < 0.01; −8% with MA30, ns) (Figure 2A). Surprisingly, only the MA30-induced decrease was not significant in our study. When the MECs were only exposed to adipose secretions (Figure 2B). The only significant effect was found with secretions of hASCs from the obese women [-22% with MC(hASC30)].

### The Adipose Microenvironment Is Able to Disrupt the Expression of the Genes Involved in the Maintenance of the Extracellular Matrix. Leptin and Inflammatory Genes Also Seem to Be Central Regulating These Disturbances

We next sought to identify the underlying mechanism by which adipose cells could act on MECs by first focusing on gene expression modifications. We investigated the modifications of the genes involved in the maintenance of the extracellular matrix (*MMP2, MMP3, MMP9, MMP14, SERPINB5, SERPINE1, FGFR1, MMP10, HSPG2, LAMA1, LAMB1, VIM, FN1*), angiogenesis (*THBS1, TIMP1, CDH1, VEGFA*), cytokine/hormonal pathways (*LEP, LEPR, ADIPOQ, ADIPOR1, ADIPOR2, ESR1, ESR2, CYP19A1*), inflammation (*IL6, TNF, PTGS2*), cell cycle/proliferation/apoptosis (*MYC, AKT1, BAX, CCND1, TP53, CTNNB1, PTEN, TGFB1, KRT5, CXCL12, MYBL2, CASP9, CCNB1, WT1, MKI67*).

**FIGURE 3:**
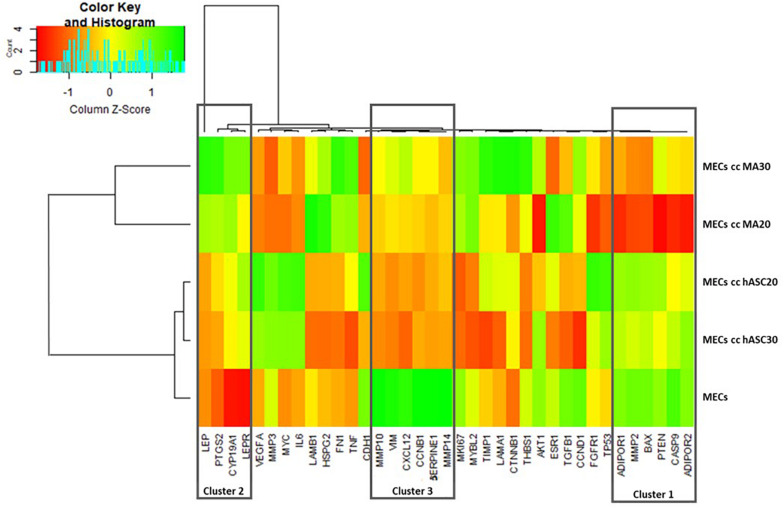
Heatmap with hierarchical clustering (red for under-expressed, yellow for unchanged expression, and green for over-expressed genes).

Hierarchical clustering enabled a clear discrimination of the MECs co-cultured, or not, with hASCs or MAs, with under-expressed genes (red font), over-expressed genes (green font) or non-modified genes (yellow font) (Figure 3). The cell type appeared to be more discriminant than the BMI of the cells. The hierarchical analysis highlighted a first cluster of genes similarly expressed in the MECs and in the MECs co-cultured with hASCs (Cluster 1: *AdipoR1, MMP2, BAX, PTEN, CASP9, AdipoR2* over-expressed in green font) compared to the cells co-cultured with MAs (red font). A second cluster presented the inverse observation, with a group of genes over-expressed in the MECs co-cultured with MAs (Cluster 2: *Leptin, leptin receptor, CYP191A1, PTGS2*). A third cluster composed of *MMP10, MMP14, vimentine, serpine1, CXCL12 and CCNB1*, could also be identified wherein their expression in the MECs is clearly up-regulated compared to the co-cultured cells, whatever the type and BMI of the adipose cells.

**FIGURE 4:**
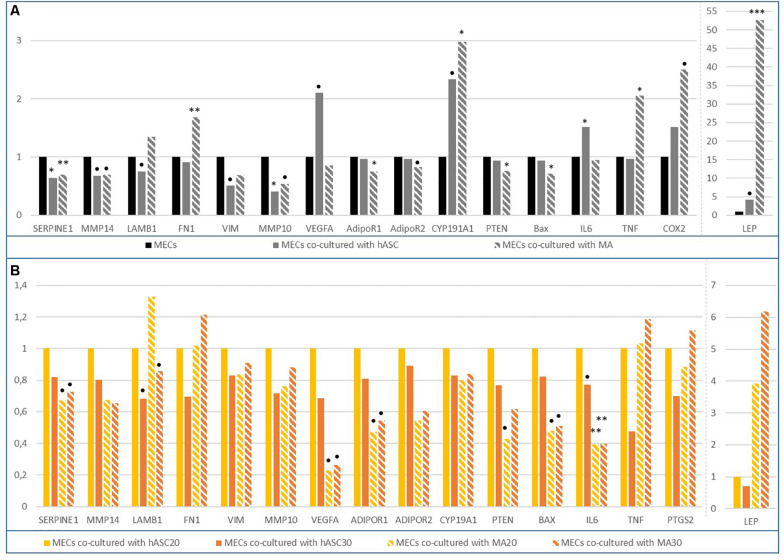
Differential gene expression in MECs co-cultured with adipose cells. **(A)** Differential gene expression in MECs co-cultured with human adipose stem cells (hASCs) or mature adipocytes (MAs); **(B)** Differential gene expression in MECs co-cultured either with hASC30 or MA20 or MA30 vs. MECs co-cultured with hASC20. ^∗^*p* < 0.05, ^∗∗^*p* < 0.01, ^∗∗∗^*p* < 0.001, *p* < 0.2.

The gene expressions were first compared between the MECs co-cultured with adipose cells, and non co-cultured cells (Figure 4A). The major role of the MECs is their involvement in the integrity of the basement membrane by producing fibronectin or laminin for example. Laminin constitutes a key element in the polarity maintenance of epithelial cells and could be a marker of MEC integrity. In our model, a tendency to decrease laminin expression was observed when MECs were co-cultivated with hASCs. The major genes involved in the maintenance of the matrix (*Serpine1, MMP14, MMP10*) were significantly under-expressed when the MECs were co-cultured with adipose cells (hASCs or MAs), except for fibronectin whose expression was increased in the presence of mature adipocytes (*RQ* = 1.7, *p* < 0.01). Among the studied ECM-degrading proteases (MMP2, 3, 9, 10, 14), the decrease in the *MMP10* gene expression when the MECs were co-cultured with hASCs, was the only observed significant modification (*RQ* = 0.4, *p* = 0.026 MECs co-cultured with hASCs vs. MECs; *RQ* = 0.5, *p* = 0.07 MECs co-cultured with MAs vs. MECs). However, for the other proteases, it is possible to note a general downward trend. When the effect of obesity was studied (Figure 4B), the expression of *LAMB1, Serpine1, MMP14, MMP10* appeared to decrease but a general downward trend could be noted.

Almost all of the studied genes involved in cell cycle, proliferation, apoptosis and even angiogenesis were not altered in the MECs co-cultured with adipose cells, suggesting that these genes were not responsible for the decrease in the viability previously observed. It seemed that the expression of tumor suppressor genes (*SerpineB5, MYC, P53, WT1, TGFB1, CCNB1, MYBL2*) were not modified, suggesting that the MECs preserve their tumor suppressor function.

On the contrary, important modifications in the expression of genes coding for cytokines/hormones or involved in inflammatory processes have been highlighted. For example, leptin expression appeared quadrupled in the MECs in the presence of adipose precursor cells (*RQ* = 4.2, *p* = 0.076) and increased by a factor of 52 with mature adipocytes (*RQ* = 52.6, *p* = 0.001) (Figure 4A). The expression was even x66 in the presence of the adipocytes from obese women vs. MECs. In parallel, the expression of AdipoR1 and AdipoR2, two adiponectin receptors, were only decreased in the MECs co-cultured with mature adipocytes. Adiponectin was not detected in our experiments. Aromatase was also stimulated by mature adipocytes (*RQ* = 2.3, *p* = 0.07 and *RQ* = 3, *p* = 0.02 for MECs co-cultured with hASCs and MAs, respectively). Interestingly, the expression of the studied inflammatory genes, *IL6, COX2*, and *TNF*, was stimulated in the MECs in the presence of adipose precursors or mature adipocytes.

Thus, adipose cells, naturally present in the tumor microenvironment, could significantly alter the gene profile of MECs by modifying the expression of the genes involved in the maintenance of the extracellular matrix. Leptin and inflammation genes also appeared to be largely impacted.

### MiR-122-5p and MiR-132-3p Could Be Targets of the Adipose Microenvironment, Leading to the Loss of the Tumor Suppressor Status of the MECs

miRNAs (or microRNAs or miRs) are small non-coding RNAs which regulate gene expression at the post-transcriptional level by inhibiting the stability or the transcriptional efficiency of target genes. Through their action of regulating the genome, miRNAs are key players in many physiopathological mechanisms, particularly cancers (He and Hannon, 2004; Yuan et al., 2019). To identify if miRNAs are involved in the modification of MEC features, we performed miRNA expression profiling analyses on the MECs co-cultured either with hASCs or MAs. Then, we looked at the influence of the BMI of the source cells.

We evaluated the expression of one of the onco-miRs which can repress tumor suppressor genes (miR-122-5p) and one of the tumor suppressor miRs which can repress oncogenes (miR-132). The onco-miR-122-5p appeared to be significantly up-regulated in MECs co-cultured with MAs compared to MECs co-cultured with hASCs (*RQ* = 2.403, *p* < 0.05) but no significant data were obtained when we looked at the BMI of the cells (Table 1). The target genes of this onco-miR were predicted by bioinformatics software (mirPath v.3 from DIANA TOOLS) which suggested, as potential bindings, a large number of genes involved in extracellular matrix maintenance, such as collagenases, integrins, laminins (*ITGB1, ITGB8, LAMB1, LAMA5, COL27A1, AGRN, LAMC3, COL4A2, COL5A1, COL4A3, CD44*). On the contrary, the expression of the tumor suppressor miR-132-3p seemed to decrease in the MECs co-cultured with MAs vs. MECs co-cultured with hASCs (*RQ* = 0.66, *p* = 0.132). A strong trend has been observed when growing with hASC30 (*RQ* = 0.479, *p* = 0.064). The target genes of this miR are, among others, involved in the TGFβ signaling pathway (*SMAD2, THBS1, ACVR2B, E2F5, SMURF1, RBL1, SMAD5, SP1, PPP2CB, MAPK1, PPP2R1B)*.

**TABLE 1:** Up- or down-regulated miRNAs in co-cultured MECs.

	**Hs cc hASCs**	**Hs cc MAs**		**Hs cc hASC20**	**Hs cc hASC30**		**Hs cc MA20**	**Hs cc MA30**	
	**Rq**	**Rq**	***P*-value**	**Rq**	**Rq**	***P*-value**	**Rq**	**Rq**	***P*-value**
hsa-miR-122-5p	1	2.403	0.039	1	0.932	0.827	1	1.782	0.314
hsa-miR-132-3p	1	0.66	0.132	1	0.479	0.064	1	0.809	0.498

### Adipose Cell Metabolome Analysis Confirmed the Specific Profiles of Each Cell Type and Their Possible Involvement in Carcinogenesis

In order to identify the molecules responsible for the effect of MEC functionality, the analysis of the culture media of hASCs and of MAs differentiated from hASCs, taken from both women of normal weight and obese women, was carried out by untargeted metabolomics.

**FIGURE 5:**
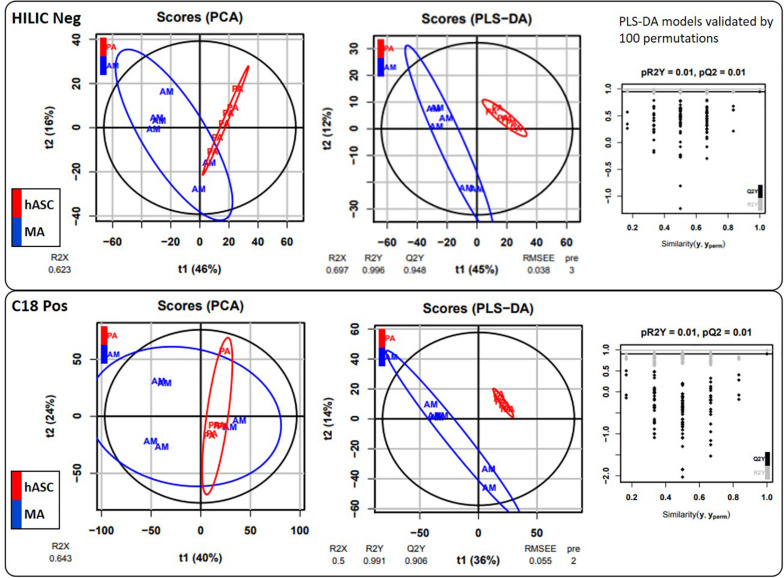
Multivariate statistical analyses. Chromatographic peak areas, contained in the two filtered and processed XCMS data matrices HILIC(−) and C18(+) (**top** and **bottom panel**, respectively), were studied using multivariate analysis (PCA, PLS-DA).

Under our conditions, 55 metabolite features from the C18(+) and 90 from the HILIC(−) analysis matched the accurate mass and retention time of the metabolites included in our chemical database, thus yielding 145 annotated metabolites in total. Differences between hASCs and MAs were investigated further using both unsupervised PCA and supervised PLS-DA analysis (Figure 5). As shown in Figure 5, PLS-DA showed a good segregation of groups, suggesting significant differences in the metabolic composition between hASCs and MAs. Permutation tests (100 times) were conducted to assess the robustness of the PLS-DA model when using a small sample size. Complementary to these multivariate analyses, an univariate analysis was applied using pairwise comparisons (hASCs vs. MAs) of individual metabolites (Wilcoxon *p*-values with Benjamini-Hochberg correction). Under these conditions, up to 31 annotated metabolites proved significant and were subjected to additional MS/MS experiments for identity confirmation (Table 2). Those metabolites belonged to several metabolic pathways, such as the BCAA metabolism (branched-chain amino acid), the antioxidant response, the nucleotide metabolism and the energy metabolism pathways. It appeared that hASCs and MAs presented different metabolic patterns characterized by an increase in the content of AA (glutamate, serine) and of free fatty acids for hASCs and, for MAs, by an increase in the content of BCAA intermediates, ketone bodies and carbohydrate metabolites. The 31 metabolites were then analyzed by the MetExplore tool to identify discriminately perturbed metabolic pathways. The following metabolic pathways proved significantly impacted: the pyrimidine catabolism pathway; the valine, leucine, and isoleucine pathways; the alanine and aspartate pathways.

**TABLE 2:** Annotated and significant metabolites.

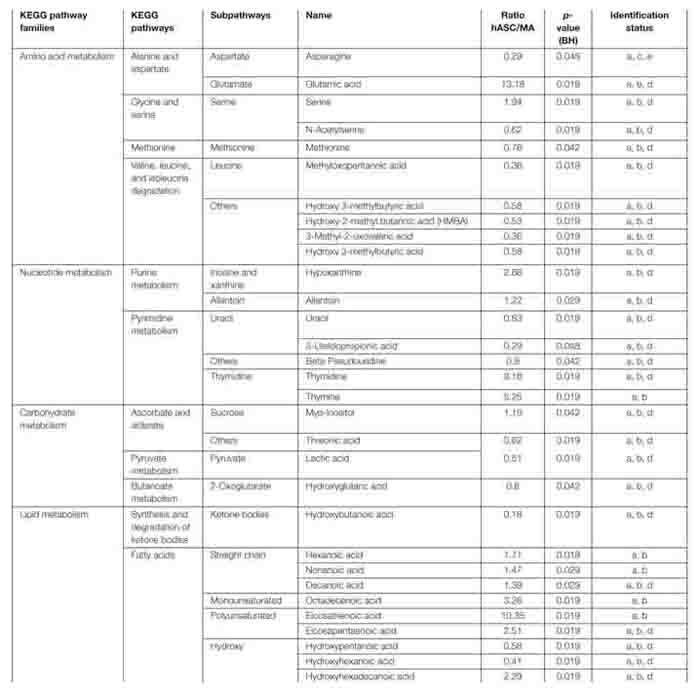

*^*a*^Based on accurate mass. ^*b*^Based on ZIC-pHILIC column retention time similarity with a standard. ^*c*^Based on C18 column retention time similarity with a standard. ^*d*^Based on MS^2^ spectrum (ESI neg) similarity with a standard.^*e*^Based on MS^2^ spectrum (ESI pos) similarity with a standard.*

## Discussion

Myoepithelial cells (MECs) are considered as tumor suppressor by regulating the functions of normal cells and by inhibiting the invasion of breast cancer cells. Studies have shown that an alteration to the functions of MECs leads to an increase in the facility of cells to migrate. So a modification to the functionality of MECs could promote carcinogenesis instead of suppressing it. Authors have hypothesized that MECs lose their tumor-suppressive features thus participating in the invasive cancer progression through the involvement of the microenvironment and the destruction of the basement membrane (Gudjonsson et al., 2002; Allinen et al., 2004; Payne et al., 2009; Lo et al., 2017). In this study, we have tried to elucidate the role of the adipose microenvironment in the modification of MEC features and wanted to evaluate if adipose cells and their secretome could modify the behavior of MECs and, consequently, be involved in the progression of cancer.

Using a co-culture model with myoepithelial cells and human adipose cells (hASCs and MAs) from women of normal weight and from obese women, we have shown that hASCs can reduce the viability of MECs, regardless of initial BMI. However, only MA20s would be able to significantly reduce the proliferation of MECs. To our knowledge, this is the first experimental evidence evaluating the impact of adipose cells on MECs in the context of breast cancer. The roles played by each cell type can probably explain the observed differences. For example, hASCs retain their ability to proliferate contrary to MAs, permitting the phenomenon of hyperplasia (Ailhaud, 1998). The different secretory activity between these two cell types is also described. hASCs are able, such as MAs, to produce some molecules, such as IGF-1, IGF-BP1,2,3, whereas the secretion of leptin is exclusively secreted by MAs (Szatkowski et al., 2013).

Our study of gene expression modifications in co-cultured MECs, in the presence of adipose cells, showed that the genes coding for the maintenance of the extracellular matrix could play a key role in the observed modifications, as did leptin and certain inflammatory genes.

One of the hallmarks of cancer is the loss of a normal tissue polarity. Our data revealed a decreased expression of Serpine-1 and an increased expression of fibronectin. During the invasive process, a lack or a dysfunction of MECs is observed. Serpine-1 (=PAI1) has been associated with breast cancer clinical outcome and is involved in the interactions between the tumor and its microenvironment, particularly in breast cancer (Vénat-Bouvet et al., 2012; Ferroni et al., 2014). In addition, Serpine-1 is known to play an important role in cell adhesion, migration and invasion (Li et al., 2018). On the contrary, Fibronectin (FN), a key component of the tumor ECM, seems to facilitate tumor formation, proliferation and angiogenesis. Its expression in breast tissue was positively correlated with an invasive and metastatic phenotype and negatively associated with survival and clinical outcome in breast cancer patients (Chen et al., 2019). Thus these two genes could constitute privileged targets for the action of adipose cells leading to the loss of MEC function. Members of the matrix metalloproteinase (MMP) family also constitute poor prognosis markers. In our study, a significant decrease in the expression of MMP14/10 was observed but, the variation was not notable for any other of the MMPs studied. Proteases are preferentially overexpressed during carcinogenesis to permit the destruction of the basement membrane and the invasion of cancer cells (Radisky and Radisky, 2015). In fact, MMPs are often produced not by epithelial cancer or myoepithelial cells but by environmental cells such as fibroblasts, macrophages (Chantrain and DeClerck, 2002) which could explain our results.

One interesting result is the impressive increase in leptin expression, particularly in the MECs co-cultured with MAs and MA30. Leptin is an adipokine whose synthesis is positively correlated with the BMI and is mainly produced by adipose cells but also by epithelial tumor cells and cells in the microenvironment, such as fibroblasts. A lot of studies have confirmed the pro-carcinogenic role of leptin through the stimulation of proliferation, migration and invasion of tumor cells (Jarde et al., 2008; Caldefie-Chézet et al., 2013; Andò et al., 2019). Thus, the adipose microenvironment could be said to play a key role in modulating the secretion of this adipokine by the MECs, which can, in turn, stimulate the proliferation of cancer cells.

Inflammation seemed to be a major biological pathway to consider. Indeed, the higher gene expression of *TNF*, *IL-6* and *COX2* in our model suggested the impact of the adipose cells. Obesity is described as associated with a chronic low-grade inflammation status characterized by the secretion of pro-inflammatory mediators (Iyengar et al., 2013). Adipose tissue produces pro-inflammatory cytokines (TNFα, TGFβ, IFNγ, IL1, IL6, IL10, IL8), chemokines (MCP1) and other biomolecules, such as PAI-1. In obese individuals, the production of these factors is altered with a balance toward pro-inflammatory factors leading to the development of low-grade systemic inflammation (Trayhurn, 2005). Low-grade chronic inflammation promotes cell proliferation through the influx of immune cells, the production of pro-inflammatory mediators and growth factors, tissue remodeling and angiogenesis (Iyengar et al., 2016).

To explain the changes in the characteristics of MECs, the other hypothesis considered here is the involvement of miRNAs. Indeed, miRNAs can be considered novel regulators in the distinctive features of human cancer (Ruan et al., 2009). A lot of studies have highlighted the involvement of miRNAs in the physiopathology of many diseases, including cancers (He and Hannon, 2004). miRNAs could be considered as tumor suppressor miRs (by repressing oncogenes) or as onco-miRs (by repressing tumor suppressor genes) according to their target gene and the tissue in which they are expressed (Zografos et al., 2019). Like any epigenetic mechanism, miRNA expression is dependent on environmental factors. In the present case, we hypothesized that the adipose microenvironment might be able to modify the expression of miRNA. In this regard, we focused on one onco-miRNA, miR-122-5p, which appeared upregulated in the MECs co-cultured with MAs. The use of dedicated bioinformatics software has enabled us to identify potential target genes involved in extracellular matrix maintenance, such as collagenases, integrins, laminins. Thus, the deregulation of this miRNA could be implicated in the significant degradation of the extracellular matrix which would no longer be regulated by the MECs. miR-122 is described as being associated with invasion and epithelial-mesenchymal transition. This miRNA seemed to be more expressed in distant metastases than in primary tumors (Uen et al., 2018) and may be a marker predicting clinical outcome of locally advanced breast cancer (Wu et al., 2012). However, some authors described miRNA as having the dual effect of tumor suppressor and oncomiR. It could promote cell survival in acquired radioresistant breast cancer (Perez-Añorve et al., 2019). On the contrary, miR-132-3p, described as a tumor suppressor miRNA, appeared down-regulated in the MECs co-cultured with MAs. Genes involved in the TGFβ signaling pathway appeared to be predicted targets of this miR, suggesting that the adipose environment could be responsible for a decrease in the MEC protective effect, favoring tumor progression. miR-132 is known as a tumor suppressor miR and some authors have associated it with reduced proliferation through its action on different targets (LAPTM4B, FOXA1) (Wang et al., 2018; Li et al., 2019).

The metabolic distinction between hASCs and MAs, particularly regarding the lipid metabolism, could also explain the observed differences in the viability assays. That’s why the identification of a cocktail of potentially bioactive molecules responsible for the described effects on the MECs has been one of our objectives. Analysis of the medium metabolome showed a strong difference between hASCs and MAs. The hASC medium was richer in glutamate, thymidine and free fatty acids and, conversely, presented a decrease in some BCAA metabolites, ketone bodies and carbohydrate metabolites. These observations suggested that, contrary to MAs, hASCs were in abundance and proliferated, resulting in the decrease of numerous metabolites implied in the synthesis of nucleotides. As well, cellular energy production was also found to be perturbed with the levels of some ketone bodies and carbohydrate metabolites being significantly impacted. In parallel, the glutamate, thymidine and free fatty acids are released in the medium and it is recognized that it might be useful to neoplastic cells as they are essential metabolites for tumor growth (O’Connell, 2013; Günther, 2015; Röhrig and Schulze, 2016). These data confirmed the roles of these two cell types described in the literature and may explain the differences observed in our co-culture assays.

Conversely, the metabolic pattern of MAs confirmed the slowdown of adipocyte proliferation but, concurrently, a change in metabolic function. The huge decrease of secreted free fatty acids highlighted the storage function of adipocytes. At the same time, it is interesting to note that, among the fatty acids identified, the two precursors of eicosanoids (dihomo gamma linoleic acid and eicosapentaenoic acid) were decreased in the medium of MAs. This observation in link with the decrease of glutamate suggested that an inflammation and antioxidant response may appear in MAs (Basu et al., 2015; Stepien et al., 2016), which we confirmed when studying gene expression in MECs (increased *IL-6*, *TNF* and *COX2* expression). In parallel, the increase in some BCAA metabolites in the environment of MAs (Leucine, isoleucine, and valine which are key regulators of protein synthesis) is in link with the metabolic switch of adipocytes and may be reinforced in an obese situation in agreement with previous data (Wang et al., 2017). The impact of BCAA on MECs has not yet been studied to our knowledge. But it is accepted that an increase in the BCAA metabolic pathway reinforces the ability of cancer cell proliferation as these metabolites are well known to be regulators of many cell signaling pathways, such as phosphorylated mTOR, S6K1, and IRS1, implied in insulin resistance and cell proliferation (O’Connell, 2013; Kogut et al., 2015; Hattori et al., 2017). Our preliminary data suggest that complementary assays should be investigated to evaluate the impact of the identified metabolites on MECs.

## Conclusion

In summary, we studied the molecular modifications of MECs surrounded by adipose cells in order to understand whether these cells and their secretome were able to disrupt the functionality of MECs and thus lead to a possible invasion of cancer cells. We have demonstrated that adipose cells, key players in the mammary tumor microenvironment, are able to act on MECs and that they could, in this way, contribute to the loss of tumor suppressor status of these cells.

## Data Availability Statement

The datasets generated for this study are publicly available. This data can be found here: MassIVE MSV000086515.

## Ethics Statement

Written informed consent was obtained from the individual(s) for the publication of any potentially identifiable images or data included in this article.

## Author Contributions

LD conducted the experiments, analyzed and interpreted the data, and wrote the manuscript. JC and AR developed the analysis methods and analyzed the data. FC and FF conducted the metabolomic experiments, interpreted the data, and wrote the manuscript. CAD and MV conducted the experiments and wrote the manuscript. CHD and CA participated in the collection of cells and interpreted the data. FCC conceptualized the study, analyzed and interpreted the data, and wrote the manuscript. All authors reviewed and approved the final version of the manuscript.

## Conflict of Interest

The authors declare that the research was conducted in the absence of any commercial or financial relationships that could be construed as a potential conflict of interest.

## Footnotes

1 https://galaxy.workflow4metabolomics.org

## Abbreviations

ADIPOQ: adiponectin
ADIPOR1: Adiponectin Receptor 1
ADIPOR2: Adiponectin Receptor 2
AKT1: AKT Serine/Threonine Kinase 1
BAX: BCL2 Associated X
BCAA: Branched-Chain Amino Acid
BMI: Body Mass Index
CASP9: Caspase 9
CCNB1: Cyclin B1
CCND1: Cyclin D1
CDH1: Cadherin 1
CI: Confidence Interval
CM: Conditioned Media
CTNNB1: Catenin Beta 1
CXCL12: C-X -C Motif Chemokine Ligand 12
CYP19A1: Cytochrome P450 Family 19 Subfamily A Member 1
DCIS: Ductal Carcinoma *In Situ*
ECM: Extra-Cellular Matrix
ER: Estrogen Receptor
ESR1: Estrogen Receptor 1
ESR2: Estrogen Receptor 2
FGFR1: Fibroblast Growth Factor Receptor 1
FN1: Fibronectin 1
GAPDH: Glyceraldehyde-3-Phosphate Dehydrogenase
GUSB: Beta-Glucuronidase
hASCs: human Adipose Stem Cells
hASC20: human Adipose Stem Cells from women of normal weight
hASC30: human Adipose Stem Cells from obese women
hMAD: human Multipotent Adipose cell line
hMAD#: human Multipotent Adipose cell differentiated in mature adipocytes
HPRT1: Hypoxanthine Phosphoribosyltransferase 1
HSPG2: Heparan Sulfate Proteoglycan 2
IDC: Invasive Ductal Cancer
IL6: Interleukin 6
KRT5: Keratin 5
LAMA1: Laminin Subunit Alpha 1
LAMB1: Laminin Subunit Beta 1
LEP: Leptine
LEPR: Leptine Receptor
MA: Mature Adipocyte
MA20: Mature Adipocyte from women of normal weight
MA30: Mature Adipocyte from obese women
MECs: MyoEpithelial Cells
MKI67: Marker of proliferation Ki 67
MMP2: Matrix Metallopeptidase 2
MMP3: Matrix Metallopeptidase 3
MMP9: Matrix Metallopeptidase 9
MMP10: Matrix Metallopeptidase 10
MMP14: Matrix Metallopeptidase 14
MYBL2: MYB Proto-Oncogene Like 2
MYC: MYC Proto-Oncogene
PTEN: Phosphatase And Tensin Homolog
PTGS2: Prostaglandin-Endoperoxide Synthase 2
RR: Relative Risk
SERPINE1: Serpin Family E Member 1
SERPINB5: Serpin Family B Member 5
TGFB1: Transforming Growth Factor Beta 1
THBS1: Thrombospondin 1
TIMP1: TIMP Metallopeptidase Inhibitor 1
TNF: Tumor Necrosis Factor
TP53: Tumor Protein P53
TP63: Tumor Protein P63
VEGFA: Vascular Endothelial Growth Factor A
VIM: Vimentin
WT1: WT1 Transcription Factor.
